# The Role of AI in Nursing Education and Practice: Umbrella Review

**DOI:** 10.2196/69881

**Published:** 2025-04-04

**Authors:** Rabie Adel El Arab, Omayma Abdulaziz Al Moosa, Fuad H Abuadas, Joel Somerville

**Affiliations:** 1 Almoosa College of Health Sciences Al Ahsa Saudi Arabia; 2 Department of Community Health Nursing, College of Nursing Jouf University Sakakah Saudi Arabia; 3 Inverness College University of the Highlands and Islands Inverness United Kingdom; 4 Glasgow Caledonian University Glasgow United Kingdom

**Keywords:** artificial intelligence, nursing practice, nursing education, ethical implications, social implications, AI integration, AI literacy, ethical frameworks

## Abstract

**Background:**

Artificial intelligence (AI) is rapidly transforming health care, offering substantial advancements in patient care, clinical workflows, and nursing education.

**Objective:**

This umbrella review aims to evaluate the integration of AI into nursing practice and education, with a focus on ethical and social implications, and to propose evidence-based recommendations to support the responsible and effective adoption of AI technologies in nursing.

**Methods:**

We included systematic reviews, scoping reviews, rapid reviews, narrative reviews, literature reviews, and meta-analyses focusing on AI integration in nursing, published up to October 2024. A new search was conducted in January 2025 to identify any potentially eligible reviews published thereafter. However, no new reviews were found. Eligibility was guided by the Sample, Phenomenon of Interest, Design, Evaluation, Research type framework; databases (PubMed or MEDLINE, CINAHL, Web of Science, Embase, and IEEE Xplore) were searched using comprehensive keywords. Two reviewers independently screened records and extracted data. Risk of bias was assessed with Risk of Bias in Systematic Reviews (ROBIS) and A Measurement Tool to Assess Systematic Reviews, version 2 (AMSTAR 2), which we adapted for systematic and nonsystematic review types. A thematic synthesis approach, conducted independently by 2 reviewers, identified recurring patterns across the included reviews.

**Results:**

The search strategy yielded 18 eligible studies after screening 274 records. These studies encompassed diverse methodologies and focused on nursing professionals, students, educators, and researchers. First, ethical and social implications were consistently highlighted, with studies emphasizing concerns about data privacy, algorithmic bias, transparency, accountability, and the necessity for equitable access to AI technologies. Second, the transformation of nursing education emerged as a critical area, with an urgent need to update curricula by integrating AI-driven educational tools and fostering both technical competencies and ethical decision-making skills among nursing students and professionals. Third, strategies for integration were identified as essential for effective implementation, calling for scalable models, robust ethical frameworks, and interdisciplinary collaboration, while also addressing key barriers such as resistance to AI adoption, lack of standardized AI education, and disparities in technology access.

**Conclusions:**

AI holds substantial promises for revolutionizing nursing practice and education. However, realizing this potential necessitates a strategic approach that addresses ethical concerns, integrates AI literacy into nursing curricula, and ensures equitable access to AI technologies. Limitations of this review include the heterogeneity of included studies and potential publication bias. Our findings underscore the need for comprehensive ethical frameworks and regulatory guidelines tailored to nursing applications, updated nursing curricula to include AI literacy and ethical training, and investments in infrastructure to promote equitable AI access. Future research should focus on developing standardized implementation strategies and evaluating the long-term impacts of AI integration on nursing practice and patient outcomes.

## Introduction

### Background

Artificial intelligence (AI) has rapidly emerged as a transformative force in health care, offering unprecedented opportunities to enhance patient care, optimize clinical workflows, and address pressing challenges, such as workforce shortages and escalating costs [1-3]. In nursing, AI applications span a broad spectrum—from predictive analytics and clinical decision support systems to web-based assistants and robotic caregivers—poised to revolutionize how nurses deliver care and interact with patients [4]. The integration of AI into nursing practice and education holds immense potential to improve health outcomes, personalize patient care, and prepare the nursing workforce for a technologically advanced health care landscape [5,6].

Despite these promising developments, the adoption of AI in nursing raises critical ethical, social, and educational concerns. Ethical challenges such as data privacy breaches, algorithmic bias, and a lack of transparency in AI decision-making processes threaten to undermine patient trust and exacerbate health disparities [7,8]. Social barriers, including resistance to change among health care professionals and unequal access to AI technologies, risk widening existing gaps in health care delivery and outcomes [9,10]. Furthermore, the current nursing education system may not be adequately preparing future nurses to navigate the complexities of AI-integrated environments, necessitating curriculum reform and the development of AI literacy programs [11,12]. There is a pressing need to equip nurses with the knowledge and skills to critically assess AI technologies, understand their limitations, and collaborate effectively with these systems [13].

AI encompasses a broad spectrum of technologies that enable machines to mimic human intelligence, including machine learning, natural language processing, robotics, and computer vision [14,15]. In health care, AI has been leveraged for tasks such as disease diagnosis, treatment planning, patient monitoring, and administrative operations [16,17].

Globally, substantial investments in AI technologies reflect a recognition of their potential to improve health care delivery. The World Health Organization emphasizes the importance of harnessing AI responsibly to achieve health for all [18]. Governments and institutions are allocating resources to develop AI infrastructure, research, and workforce training [19]. However, these developments also raise questions about the readiness of the nursing workforce to engage with AI technologies effectively and ethically.

Nurses are at the forefront of patient care, and the integration of AI into nursing practice and education represents a paradigm shift with the potential to substantially improve health care delivery. However, realizing this potential requires careful navigation of ethical challenges, social barriers, and educational needs. Therefore, it is imperative to assess AI’s integration into nursing to provide insights and evidence-based recommendations. This will support the responsible and effective adoption of AI technologies in nursing, aligning them with the professional’s core values of equity, ethics, and compassionate care.

### Rationale for an Umbrella Review

Given the proliferation of systematic, scoping, and narrative reviews evaluating AI’s impact on nursing, there is a need to consolidate these diverse findings into a single, high-level synthesis. An umbrella review integrates evidence across multiple review types [20], thus offering a broader perspective on ethical considerations, social implications, and educational practices. This approach enables us to compare existing syntheses; identify overarching gaps in literature; and provide more robust, unified recommendations for responsible AI integration in nursing practice and education. The existing literature on AI in nursing is extensive, encompassing multiple systematic, scoping, and narrative reviews that evaluate distinct aspects such as educational tools, ethical frameworks, and clinical decision support systems. However, these reviews often focus on narrow subsets of AI or specific clinical domains, making it difficult to form a comprehensive understanding of ethical and social challenges across nursing. An umbrella review synthesizes findings from multiple high-level review articles (eg, scoping reviews), thereby offering a broader perspective on common themes, methodological limitations, and evidence gaps. By integrating diverse secondary evidence, we can provide more robust, consolidated recommendations for responsible AI adoption. This approach ultimately enhances our ability to guide policy makers, educators, and practitioners in nursing toward ethically grounded and practically feasible AI integration.

### Objectives

This umbrella review aimed to evaluate the integration of AI into nursing practice and education, focusing on ethical and social implications. Our overarching aim was to provide evidence-based recommendations to support the responsible and effective adoption of AI technologies in nursing. The objectives of this umbrella review were as follows:

1. To assess the ethical and social implications of integrating AI into nursing practice and education.
2. To identify barriers influencing the adoption of AI technologies in nursing practice and education.
3. To propose evidence-based recommendations for the responsible and ethical integration of AI in nursing, ensuring alignment with core nursing values of equity, ethics, and compassionate care.

## Methods

### Study Design

This study was conducted as an umbrella review [20], designed to synthesize evidence from existing literature reviews on the integration of AI into nursing practice and education.

This approach enabled a synthesis of findings across various settings, populations, and methodologies, providing valuable insights into AI’s impact on ethical considerations, social implications, and educational strategies within the nursing profession.

This umbrella review was conducted following the PRISMA (Preferred Reporting Items for Systematic Reviews and Meta-Analyses) 2020 [21] guidelines to ensure a rigorous and transparent approach. The review process adhered to a structured methodology encompassing eligibility criteria, search strategy, screening and selection, data extraction, synthesis, and bias assessment. A thematic synthesis approach [22] was chosen to structure and interpret the findings, as it allows for the identification and organization of recurring patterns and themes across diverse datasets (PRISMA 2020 checklist is given in Multimedia Appendix 1).

### The Sample, Phenomenon of Interest, Design, Evaluation, Research Type Framework

The eligibility criteria for this umbrella review were defined using the Sample, Phenomenon of Interest, Design, Evaluation, Research type (SPIDER) tool [23], as detailed in Textbox 1. These criteria were established to capture a broad spectrum of review types that synthesize existing evidence on the integration of AI into nursing practice and education, with a focus on ethical and social implications. In this review, which examines a phenomenon (AI in nursing) rather than a clinical trial, the SPIDER tool was more appropriate than the patient or population, intervention, comparison, and outcome process framework. SPIDER was designed for qualitative and mixed methods evidence synthesis and helps define elements of a question when interventions or outcomes are not narrowly defined [23].

**Sample, Phenomenon of Interest, Design, Evaluation, Research type (SPIDER) tool components.**

**Components and description**

- S (sample): nursing professionals and nursing students
- PI (phenomenon of interest): integration of artificial intelligence (AI) into nursing practice and education, focusing on ethical and social implications
- D (design): systematic reviews, scoping reviews, rapid reviews, narrative reviews, literature reviews, and meta-analyses
- E (evaluation): ethical and social implications, barriers and facilitators to AI adoption, and the role of AI in nursing education
- R (research types): all research studies included within the reviews

### Inclusion and Exclusion Criteria

The inclusion and exclusion criteria are summarized in Textbox 2. These criteria ensured that only relevant and high-quality reviews addressing the integration of AI in nursing, particularly its ethical and social dimensions, were included.

**Inclusion and exclusion criteria.**

**Inclusion criteria**

- Publication design: systematic reviews, scoping reviews, rapid reviews, narrative reviews, literature reviews, and meta-analyses
- Focus: studies addressing the integration of artificial intelligence into nursing practice and education with emphasis on ethical and social implications.
- Population: nursing professionals, nursing students, nurse educators, and nursing researchers
- Language: publications available in English
- Time frame: studies published up to October 2024

**Exclusion criteria**

- Publication design: primary research articles, opinion pieces, editorials, conference abstracts, and gray literature
- Focus: studies not addressing ethical or social implications with reviews focusing solely on technical aspects without consideration of nursing context
- Population: studies focusing solely on medical or other health care professions without a nursing component
- Language: publications in languages other than English

### Information Sources and Search Strategy

A comprehensive literature search was conducted across multiple electronic databases to identify relevant reviews. We searched through PubMed or MEDLINE, CINAHL, Web of Science, Embase, and IEEE Xplore databases from inception up to October 2024. A new search was conducted in January 2025 to identify any potentially eligible reviews published thereafter. However, no reviews were found. These databases were selected for their broad coverage of biomedical literature, nursing research, and engineering or technology studies relevant to AI applications in health care. Search terms combined keywords and Medical Subject Headings related to “Artificial Intelligence,” and “Nursing.” The full search strategies for each database are presented in Multimedia Appendix 2.

### Selection Process

The records identified through database searches were imported into Rayyan (Rayyan Systems), a systematic review screening tool [24]. Two independent reviewers (RAEA and FHA) screened the titles and abstracts against the inclusion and exclusion criteria. Full-text articles were subsequently retrieved for studies deemed potentially relevant. Any discrepancies between reviewers were resolved through discussion or consultation with a third reviewer to reach consensus (OAAM or JS).

### Data Extraction and Analysis

In this umbrella review [20], we extracted review-level data from each included article, focusing on bibliographic details (authors, publication year, and type of review), stated objectives and scope, populations and contexts (eg, clinical nurses, nursing students, or educators), reported outcomes (ethical, social, educational, and clinical practice-related), and any limitations or recommendations noted by the review authors. Two reviewers (RAEA and FHA) conducted data extraction independently, and any discrepancies were resolved by a third reviewer (OAAM or JS) to ensure accuracy and consistency. By *high-level* reviews, we refer to systematic, scoping, and other integrative reviews that synthesize multiple primary studies.

Because our goal was to synthesize findings from existing reviews rather than reanalyze primary data, our approach centered on how each included review aggregated and interpreted evidence regarding AI integration in nursing. We used a 3-stage thematic synthesis adapted from the study by Thomas and Harden [25] to capture key concepts and recurring themes. In the first stage (open coding), 2 reviewers (RAEA and FHA) independently reviewed the extracted data, such as reported barriers, ethical concerns, or AI-driven educational strategies, to identify initial codes relevant to nursing-focused AI (eg, data privacy, algorithmic bias, curriculum reform, or clinical workflow adaptation). Next, we grouped similar codes into descriptive themes reflecting shared patterns or barriers and facilitators across the reviews. Examples of these themes include *algorithmic bias and equity*, *educational tools and AI literacy*, and *resistance to change among nurses*. Any discrepancies during this stage were resolved through consensus discussions with OAAM or JS. Finally, we developed higher-order analytical themes by linking these descriptive categories to our primary objectives: examining the ethical and social implications of AI, characterizing its transformative potential in nursing education, and proposing implementation strategies for responsible AI adoption. This interpretive phase allowed us to integrate a broad range of findings into coherent conclusions that capture the wider scope of AI-driven changes in nursing practice.

### Reflexivity and Trustworthiness

To enhance the trustworthiness of our synthesis, we maintained a detailed audit trail documenting coding decisions, the theme refinements, and conflict resolution, thereby bolstering confirmability. We addressed reflexivity [26] by regularly questioning and recording our assumptions, ensuring that thematic development was grounded in the data rather than preconceived notions. Dependability was strengthened by applying prespecified inclusion criteria aligned with the SPIDER framework and systematically noting any modifications in our search strategies or coding procedures. Credibility was reinforced through double-coding and by triangulating thematic findings with each review’s reported outcomes, enabling us to validate the synthesized themes aligned with the original evidence base [27,28]. Transferability was considered by comparing themes across various nursing contexts, including clinical, educational, and managerial settings, and across different resource levels, allowing our conclusions to be adaptable to diverse environments. By embedding these principles of rigorous qualitative synthesis [25,29,30] we aimed for a methodologically sound and transparent analysis of the included reviews, thereby accounting for the wide spectrum of perspectives on AI’s impact in nursing.

### Quality Assessment

To evaluate the methodological rigor of our included reviews, ranging from systematic and scoping to narrative and bibliometric, we used A Measurement Tool to Assess Systematic Reviews, version 2 (AMSTAR 2) [31] and the Risk of Bias in Systematic Reviews (ROBIS) tool [22]. Although both instruments were originally designed for fully systematic reviews, they encompass core elements of methodological quality that are pertinent across different review designs (eg, clarity of eligibility criteria, transparency in search methods, and coherence of data extraction). Therefore, we adapted certain domains to accommodate nonsystematic reviews that do not, for instance, conduct meta-analyses or formally appraise primary-study risk of bias. This selective, domain-level approach prevented us from unfairly penalizing reviews for lacking systematic review–specific procedures while still appraising foundational standards of rigor. By documenting these adaptations in our supplementary materials and weighing reviews’ methodological strengths and limitations accordingly, we maintained comparability across diverse review types. In practical terms, reviews meeting universal markers of transparency, such as explicit inclusion criteria or a reproducible search strategy, scored well on the relevant AMSTAR 2 and ROBIS domains, even if certain systematic-specific criteria (eg, protocol registration or meta-analysis) were not applicable. This methodology aligns with emerging best practices in synthesis research, wherein recognized tools can be judiciously tailored to evaluate the key features of any literature review.

## Results

### Overview

**Figure 1:**
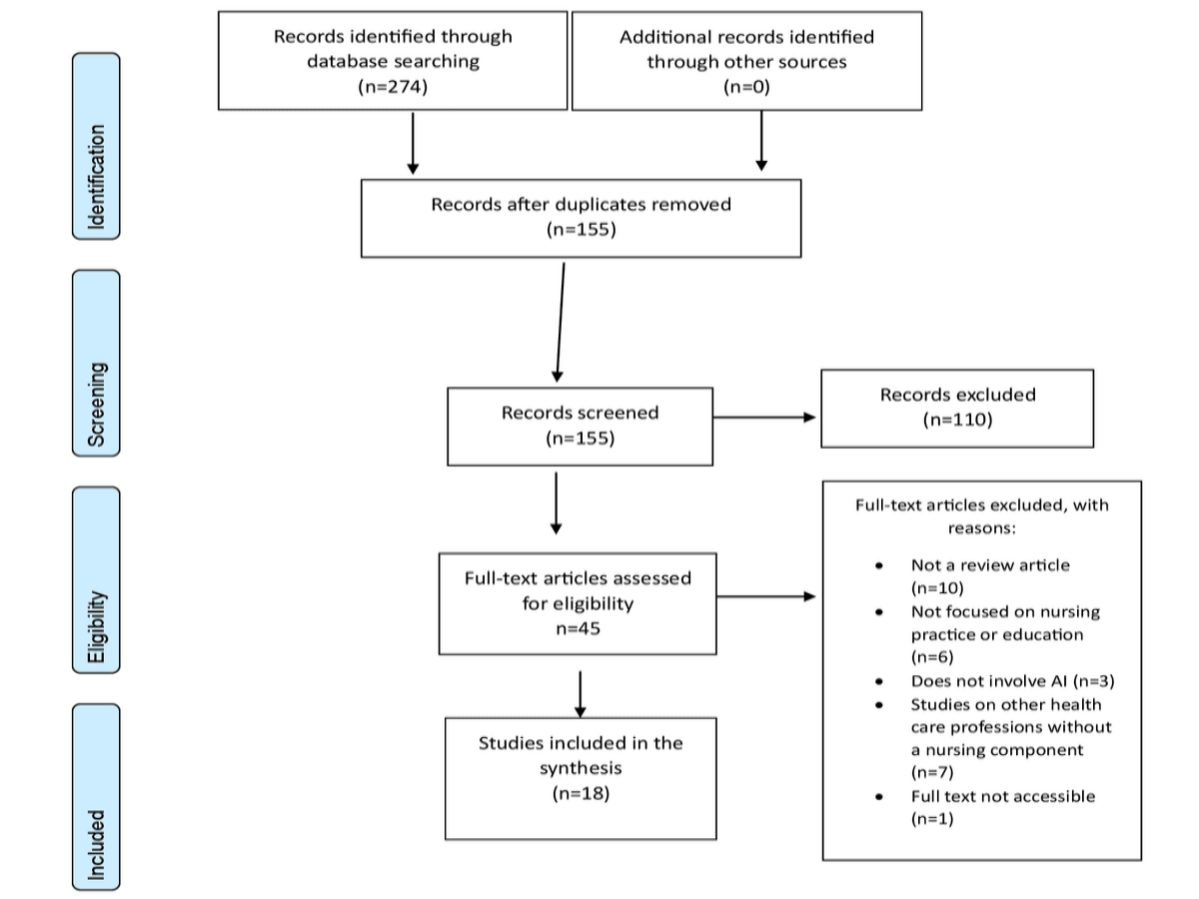
PRISMA flow diagram. AI: artificial intelligence.

The review adhered to PRISMA guidelines to ensure a transparent and systematic selection process. Initially, 274 records were identified from database searches, and after removing 43.4% (n=119) of the records as duplicates, 56.6% (n=155) of the records were unique and underwent title and abstract screening by 2 independent reviewers. This screening phase resulted in the exclusion of 71% (110/155) of records due to irrelevance, particularly those not addressing AI in nursing or lacking focus on ethical or social implications. In total, 29% (45/155) of full-text articles were then assessed in detail, with documented reasons for exclusion, including a lack of nursing focus, absence of AI-related content, inappropriate study design, and inaccessible content. Ultimately, 40% (18/45) of studies met all the inclusion criteria and were incorporated into the final synthesis, as illustrated in the PRISMA flow diagram (Figure 1).

Following our coding procedures, three major themes were identified: (1) ethical and social implications, (2) transformation of nursing education, and (3) strategies for integration. Each theme emerged from recurring patterns across the included reviews regarding data privacy, bias, curriculum integration, AI literacy, and barriers to adoption. Our interpretive phase refined these subthemes to underscore specific challenges (eg, algorithmic bias and dehumanization concerns), as well as the broader sociotechnical context (eg, interdisciplinary collaboration, policy, and equity).

### Characteristics of Included Studies

The 18 included studies in this umbrella review collectively explored the diverse applications of AI in nursing across research, practice, and education [32-49]. These studies used various methodologies, including systematic reviews, scoping reviews, rapid reviews, narrative reviews, literature reviews, and meta-analyses. The populations addressed spanned nurses, nursing students, nurse managers, educators, and researchers. The studies predominantly focused on 3 domains: research, which emphasized the development, validation, and ethical considerations of AI technologies; practice, which explored AI’s integration into workflows, decision-making, and care delivery, including predictive analytics and clinical decision support systems; and education, which examined AI’s potential in transforming nursing training through tools such as large language models (LLMs), virtual reality (VR) simulations, and personalized learning systems (Table 1 outlines the study details).

**Table 1:** Characteristics included studies in the umbrella review.

Author and year	Type of review	Objectives	Population focus	Key findings	Limitations
von Gerich et al [32], 2022	Scoping	To synthesize research on AI^a^ technologies applied in nursing, focusing on development, evaluation, and ethical considerations	Nurses in clinical settings	Highlights gaps in ethical discussions (data privacy and bias); emphasizes the need for nurse involvement in AI development; and recommends AI literacy	Majority of studies are descriptive (low-level evidence); limited real-world implementation data; and ethical discussions absent in 34 studies
Hobensack et al [33], 2024	Rapid review	To synthesize literature on current and potential applications of LLMs^b^ in nursing practice, education, and research	Nurses in practice, education, and research	Highlights the use of LLMs (eg, ChatGPT) in nursing education (- clinical practice (-), and research (-) and identifies ethical challenges, including bias, plagiarism, and privacy concerns	Most included studies are editorials or commentaries, with limited empirical research and lacks diversity in app settings beyond ChatGPT
Liu et al [34], 2023	Comprehensive review	To explore the benefits, challenges, and future directions of using ChatGPT in nursing education	Nursing students and educators	Highlights ChatGPT’s potential in personalized learning, simulation scenarios, immediate feedback, and reducing educator workload; raises concerns about accuracy, bias, and privacy	Limited empirical evidence; potential overreliance on AI; and challenges in maintaining human interaction and addressing disparities
Lifshits and Rosenberg [35], 2024	Scoping review	To explore empirical studies on AI in nursing education using a SWOT^c^ framework to assess strengths, weaknesses, opportunities, and threats.	Nursing students and educators	Identifies strengths such as improved learning experiences and enhanced critical thinking but highlights challenges, such as technical issues, language barriers, and limited realism in AI tools	Limited to studies in the English language; excluded nonempirical articles and certain nursing specializations (eg, surgical and pediatric); and methodological assessment not conducted
Montejo et al [36], 2024	Qualitative synthesis review	To review current AI applications in health care and explore implications for integrating AI in nursing education and training programs	Nursing students and educators	Identifies key AI applications (eg, predictive analytics, NLP^d^, remote monitoring, and CDSS^e^) and highlights potential in nursing education but notes ethical challenges such as bias and data security	Limited empirical studies on integrating AI education; overreliance on theoretical models; and lack of focus on diverse nursing practices
Koo et al [37], 2024	Systematic review	To investigate AI’s impact on clinical decision-making, patient care, and health care administration, with a focus on ethical considerations and regulatory frameworks	Nurses and health care practitioners	Highlights the potential of AI in improving decision-making, personalized care, and administrative efficiency and identifies ethical concerns such as data privacy, autonomy, and algorithmic bias	Lack of diverse geographical representation; focuses primarily on theoretical potential rather than real-world implementation
Ruksakulpiwat et al [38], 2024	Systematic review	To synthesize evidence on AI applications in nursing care, focusing on its impact, benefits, and challenges.	Nurses in clinical settings	Identifies 6 key themes: risk identification, health assessment, patient classification, research and development, improved care delivery, and nursing care planning and highlights ethical concerns such as privacy and bias	Limited to English-language studies; high heterogeneity in study designs and settings; and limited real-world application data
Yelne et al [39], 2023	Comprehensive review	To examine AI’s impact on nursing science and health care, addressing ethical concerns and promoting its integration	Nurses and health care professionals	Highlights the transformative potential of AI in personalized care, diagnostic accuracy, and predictive analytics and identifies ethical issues such as data privacy, bias, and the need for interdisciplinary collaboration	Limited focus on empirical studies and primarily theoretical discussion with minimal real-world applications analyzed
Mohanasundari et al [40], 2023	Comprehensive review	To examine the complementary roles of AI and human nurses in health care, focusing on empathy, decision-making, and adaptability	Nurses and health care teams	Highlights AI’s role in improving efficiency and decision-making but stresses its lack of empathy and inability to replicate the human connection inherent in nursing care	Primarily theoretical discussion with limited focus on real-world implementation and lacks empirical validation
Yasin et al [41], 2025	Scoping review	To map the roles, benefits, challenges, and future development of AI in nursing research	Nursing researchers, educators, and policy makers	Highlights benefits such as AI’s ability to enhance data analysis, predictive modeling, and literature reviews; identify barriers such as ethical challenges, methodological issues, and inequities in access	Limited focus on empirical evidence; restricted to English-language studies; and challenges in generalizing findings due to methodological diversity
Chang et al [42], 2022	Bibliometric review	To analyze AI applications in nursing research and management, focusing on academic output, research hot spots, and international collaborations	Nurses and nursing managers	Highlights AI’s potential in nursing management for decision support, quality improvement, and team communication and identifies ethical concerns, data privacy, and inequities in global adoption	Relies heavily on bibliometric methods and limited qualitative assessment of the implementation outcomes in real-world nursing settings
Seibert et al [43], 2021	Rapid review	To synthesize evidence on AI application scenarios in nursing care, focusing on ethical, legal, and social implications	Nurses, care-dependent individuals, and informal caregivers	Identifies the promise of AI in supporting clinical decision-making, care organization, and tracking health data and highlights ethical challenges such as privacy, safety, and technology acceptance	Limited real-world applications; most studies are experimental; and insufficient longitudinal data on AI’s impact on organizational or clinical outcomes
Knop et al [44], 2024	Literature review	To examine how digital technology affects nurses’ professional identity and the power dynamics within health care environments	Nurses in clinical settings	Highlights that technology can alter nurses’ identity by reducing interpersonal care; skilled nurses gain power through technology mastery; and increased dependency on technology noted	Focuses on theoretical implications with minimal empirical validation and limited to articles from the English- and German-language literature
Gonzalez-Garcia et al [45], 2024	Systematic review	To explore how AI technologies are used by nurse managers to enhance leadership, decision-making, and health care outcomes	Nurse managers in health care settings	Identifies benefits in resource management, risk assessment, and decision-making; highlights challenges such as resistance to change and ethical complexities; and advocates for targeted AI training programs	Limited empirical data and studies predominantly conceptual or early-stage with moderate to high risk of bias
Rony et al [46], 2024	Position paper and literature review	To explore the transformative role of AI in advancing nursing practice and preparing nurses for the future through education, integration strategies, and addressing ethical considerations	Nurses and nursing educators	Highlights AI’s potential to enhance clinical decision-making, reduce workload, and improve care coordination and emphasizes ethical challenges such as privacy, bias, and trust-building	Limited empirical data supporting claims; focuses primarily on conceptual discussions; no evaluation of implementation outcomes
Buchanan et al [47], 2021	Scoping review	To summarize the current and predicted influences of AI on nursing education and propose necessary curricular reforms	Nurses, nurse educators, and students	Identifies AI technologies (eg, virtual avatars and predictive analytics) transforming nursing education and highlights the need for curricula emphasizing data literacy, ethics, and AI principles	Limited empirical studies included; focuses on literature from English-language publications and gaps in real-world applications of AI in education
Li et al [48], 2024	Scoping review	To explore AI applications in psychiatric nursing, focusing on personalized care, symptom monitoring, and ethical challenges	Psychiatric nurses and patients with mental-health disorders	AI supports personalized care plans, symptom monitoring, and risk assessments and ethical concerns include data privacy, lack of transparency, and potential bias in decision-making processes	Limited focus on long-term studies; lack of diverse representation in datasets; and minimal exploration of patient perspectives on AI
O’Connor et al [49], 2024	Systematic review	To explore AI applications in cancer nursing, including its clinical impact, nurse involvement, and associated risks	Cancer nurses and patients with oncological conditions	Identified AI applications in predicting health outcomes, patient risk stratification, and care delivery and stressed the need for real-world testing and education for nurses to use AI tools effectively	Lack of clinical validation for AI tools; limited generalizability of findings due to dataset quality; and underrepresentation of susceptible populations

^a^AI: artificial intelligence.

^b^LLM: large language model.

^c^SWOT: strengths and weaknesses, opportunities and threats.

^d^NLP: natural language processing.

^e^CDSS: Clinical Decision Support System.

### Risk of Bias for Included Studies

#### Overview

The ROBIS [22] tool was selected for its relevance in assessing the methodological rigor and potential biases inherent in systematic and scoping reviews. Specifically designed to evaluate critical aspects of review methodology, such as relevance, study eligibility criteria, selection processes, data appraisal, and synthesis integrity, ROBIS provides a structured and comprehensive framework, ensuring the highest standards of assessment. This ROBIS analysis examined 18 studies (Multimedia Appendix 3 [32-49]).

#### Phase 1: Relevance

All 18 studies were deemed relevant, aligning with the umbrella review’s objectives to investigate AI’s impact on nursing practice, management, and education. The studies spanned diverse AI applications, including predictive modeling, VR, robotics, and ethical considerations, demonstrating comprehensive coverage of the subject matter.

#### Domain 1: Study Eligibility Criteria

All studies demonstrated clearly defined and rigorously applied eligibility criteria, often grounded in recognized frameworks such as PRISMA and Joanna Briggs Institute guidelines. These robust criteria enhanced the transparency and reliability of the reviews.

Systematic reviews, including the one by Gonzalez-Garcia et al [45], provided detailed eligibility justifications, strengthening the reliability of their findings. Their approach highlighted a commitment to methodological rigor and consistency.

Some reviews, such as the one by Montejo et al [36], adopted broader inclusion criteria that, while providing a comprehensive perspective, limited the depth of the insights. A more refined focus could enhance the specificity and relevance of findings.

#### Domain 2: Identification and Selection of Studies

The processes for identifying and selecting studies were generally robust, with several reviews using exhaustive multidatabase searches and citation tracking. These practices ensured the inclusion of a wide range of relevant studies and minimized the risk of publication bias.

The bibliometric analysis by Chang et al [42] and the AI in psychiatric nursing review by Li et al [48] exemplified high practices in search strategy design, capturing a broad spectrum of the literature across multiple domains.

Some reviews lacked comprehensive reporting of their search strategies, potentially limiting replicability. For example, Knop et al [44] did not fully disclose database coverage or search terms, leaving gaps in methodological transparency.

#### Domain 3: Data Collection and Appraisal

This domain exhibited variability, with several high-quality reviews using standardized data extraction and appraisal frameworks. However, inconsistencies in reporting and appraisal depth were noted in certain studies.

Gonzalez-Garcia et al [45] demonstrated meticulous attention to detail, using dual independent data extraction and rigorous appraisal methods. Similarly, Yasin et al [41] leveraged PRISMA-ScR (Preferred Reporting Items for Systematic Reviews and Meta-Analyses Extension for Scoping Reviews) guidelines to enhance reliability.

In contrast, Seibert et al [43] and Rony et al [46] provided limited details on the appraisal of included studies, raising questions about the reliability of their findings. Future efforts should prioritize comprehensive reporting of quality assessments.

#### Domain 4: Synthesis and Findings

The synthesis of findings was a strength across many studies, with coherent thematic integration and actionable insights. However, heterogeneity in methodologies occasionally posed challenges for interpretation.

Reviews such as the ones by Buchanan et al [47], Yasin et al [41], and Chang et al [42] excelled in synthesizing findings into well-defined themes, offering clear pathways for practical application.

Some studies, such as the one by O’Connor et al [49] lacked sufficient exploration of heterogeneity, which may have limited the depth of their conclusions. Future reviews should aim to contextualize findings within the broader spectrum of methodologies and outcomes.

#### Overall Assessment

The overall quality is commendable. To further elevate the impact and quality of AI research in nursing, future reviews should ensure detailed and transparent documentation of methods to enhance replicability and bolster confidence in findings. Adopting universal reporting standards such as PRISMA-S (Preferred Reporting Items for Systematic reviews and Meta-Analyses Literature Search Extension) for AI-based reviews can set a new benchmark.

### Quality Assessment of Included Studies Using AMSTAR 2

#### Overview

This umbrella review critically evaluated 18 studies on AI applications in nursing and health care using the AMSTAR 2 framework [31]. The included studies consisted of systematic reviews, scoping reviews, rapid reviews, narrative reviews, literature reviews, and meta-analyses. Each study was assessed across 8 AMSTAR 2 domains, with adaptations applied to nonsystematic review types. This evaluation provides a transparent and rigorous foundation for understanding the reliability and methodological strengths and weaknesses of the evidence base (Multimedia Appendix 4 [32-49]).

#### Adherence to Protocol Registration and Transparency

Systematic reviews such as the ones by Gonzalez-Garcia et al [45] and Ruksakulpiwat et al [38] adhered to protocol registration through platforms such as PROSPERO and Open Science Framework, demonstrating exemplary transparency. This enhances reproducibility and ensures that methods are not post hoc adjusted to fit the results. Scoping and narrative reviews, such as the one by Buchanan et al [47], followed Open Science Framework registration, while others, such as the one by Knop et al [44], did not disclose protocols.

#### Literature Search and Study Inclusion

Most systematic and scoping reviews, including the ones by O’Connor et al [49] and Yasin et al [41], conducted comprehensive and multidatabase searches. Some reviews, particularly narrative and bibliometric reviews, such as the one by Chang et al [42], lacked detailed descriptions of their search strategies, potentially missing relevant studies.

#### Study Selection and Data Extraction

Dual-review processes were widely reported in systematic reviews, ensuring independent selection and extraction. For example, Gonzalez-Garcia et al [45] and Koo et al [37] consistently used dual reviewers with conflict resolution mechanisms. In contrast, narrative reviews and rapid reviews, such as the one by Rony et al [46], often omitted these practices, which could introduce bias into their synthesis.

#### Risk of Bias Assessment

Systematic reviews such as the one by Gonzalez-Garcia et al [45], used validated tools such as Joanna Briggs Institute to assess methodological quality, offering critical insights into study reliability. Scoping reviews, such as those by Buchanan et al [47], and narrative reviews generally did not evaluate the risk of bias, consistent with their exploratory nature.

#### Heterogeneity and Synthesis

Systematic reviews, such as the one by Ruksakulpiwat et al [38] and O’Connor et al [49], acknowledged heterogeneity but often lacked systematic methods to address it. Meta-analytical methods were not applied in any of the reviews included.

Narrative and scoping review findings were typically synthesized thematically or descriptively, providing valuable insights but limited by the absence of statistical heterogeneity analyses.

#### Overall Assessment

This AMSTAR 2 evaluation revealed a dichotomy in the quality of studies on AI applications in nursing and health care. Systematic and scoping reviews adhered to rigorous methodological standards, setting benchmarks for protocol registration, structured frameworks, and comprehensive literature searches. However, deficiencies in exploratory designs, including narrative and bibliometric reviews, underscore the need for greater transparency, formal risk of bias assessments, and systematic heterogeneity analysis.

### Thematic Synthesis

#### Overview

The thematic synthesis is organized into 3 major themes: ethical and social implications, transformation of nursing education, and strategies for integration, reflecting the objectives of this review.

Table 2 summarizes the findings of the umbrella review, categorizing them into key fields, such as AI and ethics, nursing education, and clinical practice.

**Table 2:** Summary of the findings from the umbrella review categorized into key fields.

Field	Codes	References	Key findings
AI^a^ and nursing ethics	Data privacy, algorithmic bias, equitable access, accountability, and resistance to adoption	[32-34,36,37,41,43,44,46]	Persistent gaps in ethical considerations in AI implementation, including privacy and bias. Recommendations emphasize inclusive design, robust ethical frameworks, and compliance with regulations such as HIPAA^b^. Resistance stems from trust issues and fear of redundancy, emphasizing early nurse involvement in AI development. Equity concerns highlight barriers in resource-limited settings.
AI and nursing education	Curriculum integration, AI-driven tools (eg, VR^c^ and simulations), and ethical and interpersonal skill development	[34-36,40,43-47,49]	AI enhances nursing education via personalized learning and simulation platforms, but challenges include a lack of standardized curricula and overreliance on AI tools, as well as ethical concerns such as plagiarism risks and risking diminished interpersonal skills. Calls for interdisciplinary collaborations to develop comprehensive frameworks addressing ethics, equity, and technical literacy. Continuous professional development for educators remains critical.
AI in clinical practice	Clinical decision-making, predictive analytics, patient monitoring, care delivery, risk assessment, and dehumanization concerns	[36-40,43,44,46,47,49]	AI supports improved patient care through predictive modeling, personalized care plans, and real-time risk assessments. However, concerns about overreliance on technology include the erosion of compassionate care. Recommendations emphasize stakeholder involvement in AI development and addressing usability challenges.
AI in nursing management	Administrative efficiency, resource allocation, decision support, and interdisciplinary collaborations	[33,41,42,45,47]	AI reduces administrative burdens and enhances resource management. Challenges include resistance to change and ethical complexities. Collaborative approaches involving nurse managers and interdisciplinary teams are crucial for the successful integration of AI tools.
AI and interdisciplinary research	AI’s role in promoting collaboration across disciplines, facilitating literature reviews, and advancing nursing research	[33,34,41,42]	AI tools such as ChatGPT streamline research processes through enhanced data analysis and predictive modeling. Challenges include methodological diversity, ethical considerations, and equitable access to AI-driven research tools. Recommendations focus on enhancing AI literacy among researchers and ensuring datasets represent diverse populations.

^a^AI: artificial intelligence.

^b^HIPAA: Health Insurance Portability and Accountability Act.

^c^VR: virtual reality.

#### Ethical and Social Implications

##### Data Privacy and Security

The ethical and social dimensions of AI adoption in nursing were central themes across the reviewed literature, highlighting pervasive challenges related to privacy, equity, transparency, and accountability. Von Gerich et al [32] found that more than one-third (34/93, 37%) of the studies failed to address ethical considerations in AI development and deployment. Key concerns included data privacy, algorithmic bias, and their collective impact on patient autonomy. This omission reflects a systemic lack of ethical oversight in AI’s integration into health care settings, a finding echoed by Hobensack et al [33], who further detailed risks, such as the misuse of AI systems and their potential to erode critical thinking among nurses.

Bias in AI systems emerged as a critical issue, particularly in datasets that do not represent diverse populations. Liu et al [34] identified systemic biases as a cause of inequities in health care delivery, while Yasin et al [41] highlighted the risk of exacerbating existing disparities in health care access and quality. Lifshits and Rosenberg [35] contributed to this discussion by pointing out usability issues arising from language and cultural mismatches in AI tools, which hinder effective adoption, especially in diverse patient populations.

The reviewed studies consistently called for ethical frameworks that address these challenges. Recommendations included transparent accountability mechanisms, inclusive design processes that involve nurses and diverse stakeholders, and legal protections to safeguard patient data. For example, Rony et al [46] emphasized the importance of compliance with regulations such as Health Insurance Portability and Accountability Act (HIPAA) and advocated for encryption and secure data storage to protect patient information.

##### Transparency and Accountability

The lack of transparency in AI systems, often referred to as the *black box* problem, complicates accountability for errors and decisions made by these technologies. Chang et al [42] and Knop et al [44] emphasized that unclear decision-making processes undermine trust and raise ethical questions about who is liable for adverse outcomes. In addition, Ruksakulpiwat et al [38] noted a gap in regulatory frameworks to address these accountability concerns, leaving stakeholders without clear guidance on how to manage AI-related errors in clinical practice.

##### Equitable Access and Impact on Care

Equitable access to AI tools remains a substantial barrier to adoption. Von Gerich et al [32] and O’Connor et al [49] noted disparities in access to these technologies, which disproportionately affect resource-limited settings and underserved populations. This inequity threatens to widen existing gaps in health care delivery. Knop et al [44] further highlighted the dehumanizing aspects of overreliance on AI, which could erode the empathetic and interpersonal aspects of nursing care that are fundamental to the profession.

##### Resistance to Adoption and Trust Issues

Resistance to the adoption of AI among nurses was another recurring theme. Knop et al [44] highlighted skepticism about the reliability and ethical implications of AI systems. These studies underscored the importance of engaging nurses early in the development and implementation phases to foster trust and acceptance.

Koo et al [37] observed that resistance is often rooted in fear of technological redundancy, where nurses perceive AI as a threat to their professional roles.

#### Transforming Nursing Education

##### Overview

AI’s potential to reshape nursing education emerged as a central theme. The reviewed studies highlighted opportunities for integrating AI into curricula, enhancing learning experiences, and preparing nurses for technology-driven health care environments. However, challenges such as technical limitations and ethical concerns must be addressed to realize this potential fully.

##### Curriculum Integration

AI is poised to transform nursing education, but substantial gaps in preparedness and curriculum integration remain. A major barrier identified across reviews is the lack of standardized AI education and insufficient AI literacy among nurses. Von Gerich et al [32] reported that the absence of clear frameworks for integrating AI knowledge into nursing curricula has hindered progress. Similarly, Seibert et al [43] and Buchanan et al [47] emphasized the urgent need for foundational AI concepts, such as machine learning and data literacy, to be incorporated into nursing programs.

##### Enhancing Learning With AI Tools

AI-driven educational tools, including simulation platforms, VR, and chatbot-based systems, have shown promise in enhancing critical thinking, decision-making, and communication skills. For example, Lifshits and Rosenberg [35] found that VR-based learning enhanced nursing students’ ability to assess patient conditions, adapt to clinical changes, and build confidence. Similarly, Li et al [48] demonstrated how AI tools, such as wearable technologies and personalized learning platforms, support continuous skill improvement and clinical judgment. These findings were corroborated by Rony et al [46], who highlighted the value of case studies and simulations in bridging the gap between theoretical learning and practical application.

##### Ethical and Interpersonal Considerations

Despite these opportunities, risks remain. Overreliance on AI tools could undermine the development of interpersonal and empathy-driven skills, as noted by Mohanasundari et al [40]. In addition, Hobensack et al [33] raised concerns about academic integrity, such as the potential for plagiarism and the risk of students becoming overly dependent on AI-generated content. Liu et al [34] discussed the role of AI in reducing educator workload by automating routine tasks, such as grading and feedback, allowing faculty to focus on mentoring and skill development. However, they cautioned against overreliance on AI, which could undermine human interaction and empathy in education.

The ethical dimensions of AI education also require attention. Knop et al [44] argued for the integration of ethical considerations into AI education to ensure alignment with nursing values. These include reflective practices that encourage nurses to balance the technical benefits of AI with the humanistic aspects of care. O’Connor et al [49] stressed the importance of interdisciplinary collaborations between educators, health care institutions, and technology developers to create comprehensive curricula that address the multifaceted challenges of AI adoption in education.

Continuous professional development was identified as essential for equipping nurse educators with the skills needed to implement AI tools effectively. Gonzalez-Garcia et al [45] and Chang et al [42] recommended ongoing training programs to ensure that nurses remain updated on technological advancements, ethical considerations, and best practices for integrating AI into clinical education. Montejo et al [36] advocated for a multimodal educational approach that includes simulation-based learning and ethical training.

#### Strategies for Integration

##### Implementation and Scalability

Despite its transformative potential, AI integration into nursing practice and education remains in the early stages, with substantial challenges in implementation and scalability. This imbalance underscores the need for robust strategies to transition AI from experimental phases to real-world clinical and educational settings.

Automation of administrative tasks, such as documentation and scheduling, is one of AI’s most immediate benefits. Hobensack et al [33] and Ruksakulpiwat et al [38] highlighted how these capabilities reduce the burden on nurses, freeing up time for direct patient care. In addition, AI applications in clinical decision support, predictive analytics, and patient monitoring were shown to improve efficiency and patient outcomes [41,46].

Effective integration requires collaboration between key stakeholders. Buchanan et al [47] and Knop et al [44] emphasized the importance of involving nurses in the design, governance, and evaluation of AI systems to ensure alignment with clinical needs and values. Addressing usability issues is also critical.

##### Ethical Frameworks for Adoption

Ethical guidelines and standardized protocols are essential for responsible AI adoption. Chang et al [42] and O’Connor et al [49] advocated for the development of comprehensive frameworks that balance innovation with ethical considerations, ensuring that AI enhances nursing workflows without compromising patient safety or equity. Montejo et al [36] emphasized the need for clear guidelines to ensure that AI tools are used ethically, particularly in areas such as patient monitoring and decision-making. These guidelines should reflect the principles of transparency, accountability, and inclusiveness.

## Discussion

### Principal Findings

Our umbrella review synthesized 18 previous reviews covering AI in nursing practice, education, and management. Three core themes emerged: (1) ethical and social implications, underscoring persistent challenges in data privacy, bias, and transparency; (2) transformation of nursing education, highlighting gaps in AI literacy and curriculum integration; and (3) strategies for integration, emphasizing the need for ethical frameworks, stakeholder collaboration, and equitable deployment. While these findings demonstrate AI’s transformative potential, they also reveal substantial barriers—lack of standardized education, potential erosion of interpersonal skills, and ongoing inequalities in access—that must be addressed to ensure responsible and impactful AI adoption in nursing.

### Ethical and Social Implications of AI in Nursing

#### Data Privacy, Algorithmic Bias, and Accountability

The integration of AI in nursing brings substantial ethical concerns, particularly regarding data privacy, algorithmic bias, and accountability. External research highlights critical failures in these domains. For instance, Obermeyer et al [50] demonstrated racial disparities in AI-driven health care predictions, revealing how biased datasets perpetuate systemic inequities. Similarly, Zou and Schiebinger [51] identified gender biases in AI algorithms, underscoring the intersection of technology with broader social justice issues. Moreover, AI’s integration in nursing enhances diagnostic accuracy yet poses substantial ethical risks, such as data privacy breaches and algorithmic bias, that demand robust, transparent frameworks [52]. These findings suggest that the ethical challenges identified in this review reflect a global issue rather than isolated shortcomings in health care systems.

Our review revealed that while AI technologies such as predictive analytics, clinical decision support systems, and VR simulations offer substantial benefits in terms of efficiency and educational enrichment, their implementation is often hampered by resistance from nursing professionals, concerns over job displacement, and the lack of standardized ethical frameworks. The *black box* problem, where AI decision-making lacks transparency, remains a persistent challenge in high-stakes environments such as nursing. Studies, such as the one by Floridi et al [53], have highlighted the critical need for transparent and interpretable AI systems to ensure accountability. Without transparency, errors in AI predictions or decisions may lead to adverse patient outcomes without clear avenues for redress. Regulatory frameworks such as the General Data Protection Regulation and HIPAA provide essential guidelines for safeguarding patient data privacy and security, but they fall short in addressing algorithmic opacity. The FAIR (Fairness of Artificial Intelligence Recommendations) framework emphasizes independent audits, stakeholder engagement, and algorithmic accountability to ensure fairness and inclusivity [54]. This review’s findings align with these concerns, emphasizing the urgent need for legal and operational frameworks to address accountability gaps. In addition, the pervasive issue of algorithmic bias, as identified in multiple studies, highlights the urgent need for inclusive design processes and continuous monitoring to prevent the perpetuation of health care disparities.

#### Transformative Potential of AI in Nursing Education

AI-driven tools, such as LLMs, virtual simulations, and augmented reality, are redefining nursing education by enhancing critical thinking, clinical judgment, and personalized learning experiences. However, the lack of standardized AI literacy programs and insufficient integration of AI concepts into nursing curricula limit the preparedness of future nurses. Risks of overreliance on AI tools and potential erosion of interpersonal skills also warrant careful consideration. AI-driven tools boost clinical outcomes and streamline operations, yet the absence of standardized AI literacy programs risks overreliance and erosion of essential interpersonal skills [52].

Developing structured curricula that incorporate foundational AI concepts, ethical principles, and practical simulations is essential. Educator training programs should also be prioritized to enable confident and responsible use of AI in academic and clinical settings [43,47]. Furthermore, embedding ethical considerations into nursing curricula and establishing robust regulatory frameworks are imperative to safeguard patient autonomy, data privacy, and the integrity of nursing care [44,46].

#### Transformative Potential and Strategies for AI Integration in Nursing Practice and Education

AI is reshaping the landscape of nursing practice and education, offering transformative opportunities to enhance care delivery, decision-making, and learning experiences. However, the findings of this review underscore substantial gaps in implementation and operationalization, ethical governance, equity, and curriculum integration. Addressing these challenges requires a holistic approach that prioritizes technological advancement alongside humanistic and ethical considerations.

AI’s potential to revolutionize nursing education is substantial, offering opportunities to enhance learning experiences and preparing nurses for a technologically advanced health care environment. However, the integration of AI into curricula lacks standardization.

AI-driven educational tools, such as simulation platforms and VR, have shown promise in enhancing critical thinking and clinical decision-making skills, aligning with findings from Foronda et al [55], who reported that simulation technologies enhance clinical judgment. These similarities reinforce the potential benefits of incorporating AI tools into nursing education.

Despite these opportunities, concerns about overreliance on AI and the potential erosion of interpersonal skills persist. A concern was also raised by Shorey and Ng [56], who argued that VR technological advancements should not compromise the humanistic aspects of nursing. The alignment of these findings suggests a need for balanced integration that preserves core nursing values. Furthermore, continuous professional development was identified as essential for equipping nurse educators with the skills needed to implement AI tools effectively [42,45].

### Strategies for Effective AI Integration in Nursing Practice

#### Strategies for Effective AI Integration in Nursing Practice: Bridging the Gap Between Development and Operationalization

Despite substantial advancements in AI research, the transition from development to operationalization remains a critical challenge. Although AI technologies—including predictive analytics, clinical decision support systems, and workflow automation—offer great potential for nursing practice, the literature shows a disproportionate emphasis on early-stage development, with only a limited number of studies addressing implementation and even fewer exploring operationalization [32]. This disconnect hinders the realization of AI’s transformative potential in real-world nursing environments, where systemic barriers such as financial constraints, infrastructure readiness, and workforce training remain inadequately addressed [39,45].

Practical strategies that leverage AI’s capabilities can offer immediate benefits to nursing practice. Automating administrative tasks such as documentation, scheduling, and resource allocation not only reduces nurse workload but also enhances workflow efficiency and patient safety [45,57]. Similarly, AI-driven predictive analytics and clinical decision support systems contribute to optimized patient outcomes by enabling early intervention and personalized care strategies [32,45]. These tools exemplify how AI can augment nursing practice, if implementation frameworks account for the unique challenges of diverse health care contexts [57].

In nursing education, AI technologies are redefining how students acquire and apply clinical skills. Tools such as LLMs, augmented reality, and VR offer immersive learning environments that enhance clinical judgment, critical thinking, and decision-making capabilities [32,36]. By simulating realistic scenarios, these technologies bridge the gap between theoretical knowledge and practical application, allowing students to refine their skills in controlled, feedback-rich settings [36,47]. Moreover, real-time AI-driven feedback systems support continuous skill improvement, ensuring that nursing graduates are well-prepared for the complexities of modern health care [47].

However, systemic challenges persist. The absence of standardized AI literacy programs and insufficient integration of AI concepts into nursing curricula limit the preparedness of future nurses to engage effectively with these technologies [39,47]. This gap risks fostering overreliance on AI systems, potentially eroding essential interpersonal skills and undermining core nursing values such as empathy and patient-centered care [45,57]. Furthermore, the lack of real-world implementation data, particularly from pilot programs and case studies, leaves critical questions unanswered regarding how AI can be sustainably and equitably operationalized in resource-limited settings [32].

Addressing these gaps requires a strategic shift in focus. Future research should prioritize the development of adaptable frameworks that guide the integration of AI into nursing practice and education. These frameworks must consider not only the technical capabilities of AI but also the systemic and infrastructural changes required to support equitable and sustainable implementation. Effective AI integration in nursing hinges on interdisciplinary collaboration and longitudinal research, especially in resource-limited settings, to ensure sustainable and equitable implementation [52].

#### Promoting Equity and Accessibility in AI Deployment

AI adoption is marred by disparities in access, particularly in resource-limited settings. Inclusive policy frameworks, as advocated by Wachter and Cassel [58], are essential to bridge these gaps. Investments in localized AI deployment strategies, such as culturally sensitive designs and infrastructure development in underserved regions, can ensure that the benefits of AI are equitably distributed. Moreover, the pervasive issue of algorithmic bias, as identified in multiple studies, highlights the urgent need for inclusive design processes and continuous monitoring to prevent the perpetuation of health care disparities [34,41]. Ensuring equitable access to AI tools remains a pressing concern, and regulatory frameworks must address these disparities to promote fairness and inclusivity in AI applications [42,45].

From a sociotechnical perspective [59], successful AI integration in nursing requires alignment between technological tools, the organizational context in which they are deployed, and the nurses who use them. This echoes the Technology Acceptance Model [60], which posits that perceived usefulness and ease of use critically influence adoption. By integrating sociotechnical considerations, such as workflow compatibility, nurse involvement in AI design, and ethical guardrails, future implementations are more likely to foster positive attitudes, reduce resistance, and ultimately support the ethical, patient-centered use of AI in nursing practice.

### Strengths and Limitations

This umbrella review provides a comprehensive and critical synthesis of the current literature on the ethical, social, and educational implications of AI in nursing. By systematically evaluating 18 studies through established assessment tools ROBIS and AMSTAR 2, we ensured methodological rigor and transparency. Our adherence to recognized frameworks enhanced the reliability and reproducibility of our findings.

One of the key strengths of this review lies in its holistic approach. By encompassing a wide range of study designs—including systematic reviews, scoping reviews, rapid reviews, and bibliometric analyses—we captured diverse perspectives on AI’s impact across nursing practice, education, and research. This inclusivity allowed us to identify common themes and gaps, providing a multifaceted understanding of the challenges and opportunities presented by AI integration in nursing.

Our thematic synthesis was meticulously organized into 3 major themes: ethical and social implications, transformation of nursing education, and strategies for integration. By highlighting issues such as data privacy, algorithmic bias, curriculum integration, and ethical governance, we offer insights that are directly applicable to policy makers, educators, and practitioners.

Despite these strengths, our umbrella review has limitations that warrant consideration. This umbrella review synthesized findings from diverse review types, including systematic, scoping, and narrative reviews, each using varied methodologies and addressing heterogeneous populations and settings. While this diversity provides a broad perspective on the integration of AI into nursing, it also poses challenges for cohesive synthesis and comparability. The lack of quantitative analyses, inherent to umbrella reviews that exclude individual studies, further limits the ability to quantitatively assess outcomes. These findings underscore the need for more standardized methodologies and reporting practices in future reviews to enable stronger synthesis and actionable conclusions.

Another limitation is the potential for publication bias. Our reliance on published literature reviews may have excluded relevant studies available only in individual studies, gray literature, or non-English languages, potentially skewing our findings. Although some reviews included attempts to mitigate this by incorporating gray literature, the overall effect cannot be fully ascertained.

### Recommendations and Implications

#### Establish Comprehensive Ethical Frameworks

Developing robust regulatory guidelines is paramount to ensuring responsible AI adoption in nursing practice. Such guidelines should extend beyond established frameworks such as General Data Protection Regulation and HIPAA, considering the unique challenges posed by AI-driven clinical decisions, algorithmic biases, and data privacy. Regular audits for fairness and security are equally essential. By mandating periodic checks for algorithmic integrity and data protection, health care institutions can detect and address biases early, thus safeguarding both patient welfare and public trust.

#### Integrate AI Education Into Nursing Curricula

Preparing future nurses for an increasingly technology-driven environment requires a systematic revision of nursing curricula to incorporate foundational AI concepts, data literacy, and practical ethical training. This approach empowers nurses to critically evaluate AI-generated outputs and to collaborate effectively with advanced digital tools. Equally important is faculty development: providing educators with the knowledge and confidence to integrate AI-based teaching methods not only enhances learning outcomes but also fosters a workforce adept at navigating complex digital landscapes.

#### Foster Interdisciplinary Collaboration

Promoting partnerships among nurses, data scientists, ethicists, and engineers encourages the design and implementation of AI systems that are ethical, user-centered, and clinically relevant. By including patient and community representatives in these discussions, developers gain insights into linguistic, cultural, and socioeconomic nuances that might otherwise be overlooked. This collaborative dynamic cultivates AI solutions that are not only technologically sound but also equitable and broadly acceptable.

#### Promote Transparency and Accountability

Enhancing the interpretability of AI models is vital for building trust among nurses, patients, and decision-makers. Algorithms capable of explaining their decision pathways help ensure that clinical judgments remain transparent and can be scrutinized for biases or errors. Beyond transparency, defining clear protocols for handling AI-related adverse events establishes accountability and reinforces patient safety. When responsibilities are delineated at organizational and professional levels, it becomes clearer how to address unintended consequences and system failures.

#### Address Equity and Accessibility

Substantial investments are needed to extend AI technologies to underserved regions, ensuring that socioeconomic constraints do not widen the existing digital divide. By deploying AI tools that are culturally tailored, accommodating linguistic and demographic variations, health care delivery can become more inclusive. In this way, AI can serve as a powerful lever for enhancing rather than exacerbating health equity, particularly when implemented with deliberate planning and resource allocation.

#### Encourage Continuous Professional Development

Ongoing education and training initiatives enable practicing nurses to stay abreast of cutting-edge AI tools, emerging data management techniques, and shifting ethical standards. Designing accessible modules, whether web-based or blended learning, can promote widespread engagement. Moreover, creating incentives and funding for nurse-led AI research helps spawn innovative pilot programs that link theoretical advancements to concrete clinical outcomes, fostering a cycle of continual improvement.

#### Cultivate Ethical Leadership in Nursing

Nurses have long been trusted figures in health care and positioning them as leaders on AI-related boards, committees, and decision-making panels ensures that ethical considerations remain central to technological progress. Strengthening ethics education that prioritizes patient autonomy, consent, and relational care enables nurse leaders to critically assess both the potential and the pitfalls of AI. By uniting professional values with forward-looking strategies, nursing professionals can guide AI initiatives that enhance patient care while preserving the core compassion of the discipline.

### Conclusions

This umbrella review comprehensively explored the integration of AI into nursing practice and education. However, the adoption of AI in nursing is fraught with critical ethical and social challenges. Key concerns include data privacy breaches, algorithmic bias, and a lack of transparency and accountability in AI-driven decisions. To address these challenges, a strategic and holistic approach is essential. This involves establishing comprehensive ethical frameworks and robust regulatory guidelines to safeguard patient data and ensure algorithmic fairness. In addition, integrating AI literacy into nursing curricula is crucial to prepare future nurses with the necessary knowledge and skills to effectively navigate technology-driven environments. Fostering interdisciplinary collaboration, continuous professional development, and inclusive policies should be prioritized to support ongoing adaptation and responsible AI adoption. Future research should focus on evaluating the long-term impacts of AI integration and developing standardized implementation strategies to facilitate its responsible adoption in health care.

## Appendix

Multimedia Appendix 1 PRISMA (Preferred Reporting Items for Systematic Reviews and Meta-Analyses) checklist.

Multimedia Appendix 2 Complete database search strategies.

Multimedia Appendix 3 Risk of Bias in Systematic Reviews (ROBIS) for the included reviews.

Multimedia Appendix 4 A Measurement Tool to Assess Systematic Reviews (AMSTAR 2) quality assessment for the included reviews.

## Abbreviations

AI: artificial intelligence
AMSTAR 2: A Measurement Tool to Assess Systematic Reviews, version 2
HIPAA: Health Insurance Portability and Accountability Act
LLM: large language model
PRISMA: Preferred Reporting Items for Systematic Reviews and Meta-Analyses
PRISMA-S: Preferred Reporting Items for Systematic reviews and Meta-Analyses Literature Search Extension
PRISMA-ScR: Preferred Reporting Items for Systematic Reviews and Meta-Analyses Extension for Scoping Reviews
ROBIS: Risk of Bias in Systematic Reviews
SPIDER: Sample, Phenomenon of Interest, Design, Evaluation, Research type
VR: virtual reality

## Data Availability

All data generated or analyzed during this study are included in this published article and its supplementary information files.
