# Supplementary material for: The Role of AI in Nursing Education and Practice: Umbrella Review
Source: J Med Internet Res. 2025 Apr 4;27:e69881. doi: 10.2196/69881 (PMC12008698; doi:10.2196/69881)
Supplement: Multimedia Appendix 2 [file jmir_v27i1e69881_app2.docx]

**Appendix A: Complete Database Search Strategies**

| Database | Search String | Limits / Comments |
| --- | --- | --- |
| PubMed/MEDLINE | (("Artificial Intelligence"[Mesh] OR "Artificial Intelligence" OR "AI" OR "Machine Learning" OR "Deep Learning" OR "Natural Language Processing" OR "Neural Networks") AND ("Nursing"[Mesh] OR "Nursing" OR "Nurse" OR "Nursing Practice" OR "Nursing Education" OR "Nursing Students" OR "Nursing Researchers") AND ("Ethics"[Mesh] OR "Ethical Considerations" OR "Ethical Implications" OR "Data Privacy" OR "Algorithmic Bias" OR "Social Implications" OR "Barriers" OR "Facilitators" OR "Adoption" OR "Implementation")) AND (Review[ptyp] OR "systematic review" OR "scoping review" OR "rapid review" OR "literature review" OR "meta-analysis") | - Language: English  - Publication Types: Review, Systematic Review, Scoping Review, Rapid Review, Narrative Review, Meta-Analysis - Date Range: Inception to October 202 |
| CINAHL | ("Artificial Intelligence" OR "Machine Learning" OR "Deep Learning" OR "Natural Language Processing" OR "Neural Networks") AND ("Nursing" OR "Nursing Education" OR "Nursing Students" OR "Nurse*") AND ("Ethics" OR "Ethical" OR "Algorithmic Bias" OR "Data Privacy" OR "Social Implications" OR "Barriers" OR "Facilitators" OR "Resistance to Change")) AND (MH "Review" OR "Systematic Review" OR "Scoping Review" OR "Meta Analysis") | Peer Reviewed - English Language - Subject Headings (CINAHL) |
| Web of Science | TS=((("Artificial Intelligence") OR ("Machine Learning") OR ("Deep Learning") OR ("Natural Language Processing") OR ("Neural Network")) AND (Nurs* OR "Nursing Education" OR "Nursing Practice") AND ("Ethical Considerations" OR "Ethical Implications" OR "Social Implications" OR "Algorithmic Bias" OR "Data Privacy" OR "Barriers" OR "Facilitators" OR "Adoption")) AND TS=(Review OR "Systematic Review" OR "Scoping Review" OR "Meta-Analysis" OR "Narrative Review") | - Language: English  - Publication Types: Review, Systematic Review, Scoping Review, Rapid Review, Narrative Review, Meta-Analysis - Date Range: Inception to October 202 |
| Embase | ('artificial intelligence'/exp OR 'machine learning'/exp OR 'deep learning'/exp OR 'natural language processing'/exp OR 'neural network'/exp) AND ('nursing'/exp OR 'nurse'/exp OR 'nursing education'/exp OR 'nursing student'/exp) AND ('ethics'/exp OR 'ethical consideration' OR 'algorithmic bias' OR 'data privacy' OR 'barriers' OR 'facilitators' OR 'adoption'/exp) AND ([review]/lim OR [meta analysis]/lim OR [systematic review]/lim OR [scoping review]) | - Language: English  - Limit to: Review-type articles |
| IEEE Xplore | ((("Artificial Intelligence") OR ("Machine Learning") OR ("Deep Learning") OR ("Natural Language Processing") OR ("Neural Networks")) AND ((Nursing) OR ("Nursing Education") OR ("Nurse"))) AND (("Ethics") OR ("Ethical") OR ("Algorithmic Bias") OR ("Data Privacy") OR ("Social Implications") OR ("Barriers") OR ("Facilitators") OR ("Implementation")) AND (("Review") OR ("Systematic Review") OR ("Scoping Review") OR ("Meta-Analysis")) | Content Type: Journals & Conferences  - Language: English  - Date Range: Up to October 2024 |
