# Supplementary material for: The Role of AI in Nursing Education and Practice: Umbrella Review
Source: J Med Internet Res. 2025 Apr 4;27:e69881. doi: 10.2196/69881 (PMC12008698; doi:10.2196/69881)
Supplement: Multimedia Appendix 4 [file jmir_v27i1e69881_app4.docx]

**Appendix C: AMSTAR 2 Quality Assessment for the included reviews**

| Study | Overall Quality | Key Strengths | Key Limitations |
| --- | --- | --- | --- |
| Von Gerich et al [32] | Moderate | Protocol registration, comprehensive search | No bias assessment |
| Hobensack et al [33] | Moderate | Protocol registration, use of OSF, duplicate selection | No systematic heterogeneity analysis |
| Liu et al [34] | Critically Low | Exploratory insights into ChatGPT | No protocol registration or bias assessment |
| Lifshits & Rosenberg [35] | Low | Use of PCC framework, PRISMA adherence | No protocol registration, no bias assessment |
| Montejo et al [36] | Low | Exploratory perspective on nursing education | No protocol registration or systematic framework |
| Koo et al [37] | High | PICO framework, bias assessment, comprehensive search | Limited heterogeneity analysis |
| Ruksakulpiwat et al [38] | High | PRISMA adherence, robust bias analysis | Limited details on heterogeneity impacts |
| Yelne et al [39] | Critically Low | Broad insights into AI applications | No protocol registration or bias analysis |
| Mohanasundari et al [40] | Critically Low | Nuanced exploration of nursing roles | No protocol registration, lack of framework |
| Yasin et al [41] | Moderate | PCC framework, detailed thematic analysis | No risk of bias assessment |
| Chang et al [42] | Critically Low | Exploration of AI trends in nursing | Lack of structured framework, no protocol |
| Seibert et al [43] | Low | Rapid synthesis of evidence | Single-reviewer full-text screening |
| Knop et al [44] | Low | Impact of digital tech on identity | No protocol registration, no heterogeneity |
| Gonzalez-Garcia et al [45] | High | Quantitative and qualitative synthesis | Limited heterogeneity analysis |
| Rony et al [46] | Critically Low | Position paper with conceptual themes | No bias assessment, thematic focus |
| Buchanan et al [47] | Moderate | Comprehensive education insights | No bias assessment, descriptive themes |
| Li et al [48] | Low | Mapping psychiatric AI applications | No protocol, limited transparency |
| O’Connor et al [49] | High | PEOS framework, robust evidence hierarchy | No detailed heterogeneity exploration |

Note: AMSTAR 2 was adapted to each review’s methodology, evaluating only the relevant domains for scoping, narrative, and bibliometric reviews. Criteria specific to fully systematic reviews, such as meta-analysis requirements, were omitted where inapplicable. Refer to “Quality Assessment” in Methods for further explanation.

**References**

[32] von Gerich H, Moen H, Block LJ, Chu CH, DeForest H, Hobensack M, et al. Artificial Intelligence -based technologies in nursing: A scoping literature review of the evidence. Int J Nurs Stud 2022;127:104153. https://doi.org/10.1016/J.IJNURSTU.2021.104153.

[33] Hobensack M, von Gerich H, Vyas P, Withall J, Peltonen LM, Block LJ, et al. A rapid review on current and potential uses of large language models in nursing. Int J Nurs Stud 2024;154:104753. https://doi.org/10.1016/J.IJNURSTU.2024.104753.

[34] Liu J, Liu F, Fang J, Liu S. The application of Chat Generative Pre-trained Transformer in nursing education. Nurs Outlook 2023;71. https://doi.org/10.1016/j.outlook.2023.102064.

[35] Lifshits I, Rosenberg D. Artificial intelligence in nursing education: A scoping review. Nurse Educ Pract 2024;80:104148. https://doi.org/10.1016/J.NEPR.2024.104148.

[36] Montejo L, Fenton A, Davis G. Artificial intelligence (AI) applications in healthcare and considerations for nursing education. Nurse Educ Pract 2024;80:104158. https://doi.org/10.1016/J.NEPR.2024.104158.

[37] Koo TH, Zakaria AD, Ng JK, Leong X Bin. Systematic Review of the Application of Artificial Intelligence in Healthcare and Nursing Care. Malays J Med Sci 2024;31:135–42. https://doi.org/10.21315/MJMS2024.31.5.9.

[38] Ruksakulpiwat S, Thorngthip S, Niyomyart A, Benjasirisan C, Phianhasin L, Aldossary H, et al. A Systematic Review of the Application of Artificial Intelligence in Nursing Care: Where are We, and What&rsquo;s Next? J Multidiscip Healthc 2024;17:1603–16. https://doi.org/10.2147/JMDH.S459946.

[39] Yelne S, Chaudhary M, Dod K, Sayyad A, Sharma R. Harnessing the Power of AI: A Comprehensive Review of Its Impact and Challenges in Nursing Science and Healthcare. Cureus 2023;15. https://doi.org/10.7759/CUREUS.49252.

[40] Mohanasundari SK, Kalpana M, Madhusudhan U, Vasanthkumar K, B R, Singh R, et al. Can Artificial Intelligence Replace the Unique Nursing Role? Cureus 2023;15. https://doi.org/10.7759/CUREUS.51150.

[41] Yasin YM, Al-Hamad A, Metersky K, Kehyayan V. Incorporation of artificial intelligence into nursing research: A scoping review. Int Nurs Rev 2024. https://doi.org/10.1111/INR.13013.

[42] Chang CY, Jen HJ, Su WS. Trends in artificial intelligence in nursing: Impacts on nursing management. J Nurs Manag 2022;30:3644–53. https://doi.org/10.1111/JONM.13770.

[43] Seibert K, Domhoff D, Bruch D, Schulte-Althoff M, Fürstenau D, Biessmann F, et al. Application Scenarios for Artificial Intelligence in Nursing Care: Rapid Review. J Med Internet Res 2021;23(11):E26522 Https://WwwJmirOrg/2021/11/E26522 2021;23:e26522. https://doi.org/10.2196/26522.

[44] Knop M, Mueller M, Kaiser S, Rester C. The impact of digital technology use on nurses’ professional identity and relations of power: a literature review. J Adv Nurs 2024;80:4346–60. https://doi.org/10.1111/JAN.16178.

[45] Gonzalez-Garcia A, Pérez-González S, Benavides C, Pinto-Carral A, Quiroga-Sánchez E, Marqués-Sánchez P. Impact of Artificial Intelligence–Based Technology on Nurse Management: A Systematic Review. J Nurs Manag 2024;2024:3537964. https://doi.org/10.1155/2024/3537964.

[46] Rony MKK, Parvin MR, Ferdousi S. Advancing nursing practice with artificial intelligence: Enhancing preparedness for the future. Nurs Open 2024;11. https://doi.org/10.1002/NOP2.2070.

[47] Buchanan C, Howitt ML, Wilson R, Booth RG, Risling T, Bamford M. Predicted Influences of Artificial Intelligence on Nursing Education: Scoping Review. JMIR Nurs 2021;4:e23933. https://doi.org/10.2196/23933.

[48] Li H, Zhu G, Zhong Y, Zhang Z, Li S, Liu J. Applications of Artificial Intelligence in Psychiatric Nursing: A Scope Review. Stud Health Technol Inform 2024;315:74–80. https://doi.org/10.3233/SHTI240109.

[49] O’Connor S, Vercell A, Wong D, Yorke J, Fallatah FA, Cave L, et al. The application and use of artificial intelligence in cancer nursing: A systematic review. Eur J Oncol Nurs 2024;68. https://doi.org/10.1016/J.EJON.2024.102510.
